# Impact of Simulated Human Gastrointestinal Digestion on the Bioactive Fraction of Upcycled Pineapple By-Products

**DOI:** 10.3390/foods11010126

**Published:** 2022-01-05

**Authors:** Ricardo Gómez-García, Ana A. Vilas-Boas, Ana Oliveira, Manuela Amorim, José A. Teixeira, Lorenzo Pastrana, Maria Manuela Pintado, Débora A. Campos

**Affiliations:** 1 CBQF—Centro de Biotecnologia e Química Fina—Laboratório Associado, Escola Superior de Biotecnologia, Universidade Católica Portuguesa, 4169-005 Porto, Portugal; rgarcia@ucp.pt (R.G.-G.); avboas@ucp.pt (A.A.V.-B.); asoliveira@ucp.pt (A.O.); mamorim@ucp.pt (M.A.); dcampos@ucp.pt (D.A.C.); 2 Centro de Engenharia Biológica, Universidade do Minho—Campus Gualtar, 4710-057 Braga, Portugal; jateixeira@deb.uminho.pt; 3 INL—International Iberian Nanotechnology Laboratory, 4710-330 Braga, Portugal; lorenzo.pastrana@inl.int

**Keywords:** pineapple by-products, prebiotic activity, antioxidant activity, bioactive compounds, gastrointestinal tract simulation

## Abstract

Pineapple by-products (peels and stems) from fruit processing industries were evaluated to understand its potential application as a functional food. Therefore, the bioactive compounds of pineapple by-products were characterized for prebiotic and antioxidant activities. A total characterization of soluble carbohydrates profile (simples and complex carbohydrates), as well as polyphenols was performed, after removal of enzymatic fraction from pineapple crude juice, allowing the decrease of proteolytic activity and improving the other biological activities. Results showed that pineapple liquid fraction, from stem and peels, can be applied as a prebiotic enhancer, promoting the growth of five probiotic microorganisms (two strains of *Lactobacillus* sp. and three strains of *Bifidobacterium* sp.), as a single carbohydrate source. Moreover, through HPLC (High Performance Liquid Chromatography) analysis, 10 polyphenols were identified in pineapple liquid fractions, with some expected differences between both evaluated by-products. Gastrointestinal tract was simulated, in a continuous mode to understand the impact of pH changes and gastrointestinal enzymes into pineapple liquid fractions. Results showed a digestion of high molecular weight polysaccharides into small molecular weight tri-, di-, and monosaccharides. There was an increase of samples antioxidant activity through the gastrointestinal stage, followed by the release of specific polyphenols, such as chlorogenic, coumaric, and ferulic acids. The prebiotic activity did not improve throughout the simulation, in fact, the prebiotic potential decreased throughout the different stages.

## 1. Introduction

Food waste management within the food industry is critical, stimulating increasing interest into the development of new valorization strategies. Industries that produce juices, canned food, and dehydrated snacks from fruit and vegetables deals with waste production every day and often these are infra-used as feed or fertilizer [1]. Several strategies have arisen for more efficient, low-cost, and environmentally friendly uses of these materials and have become more important to the scientific field [2], and has been a challenge that must be addressed to a more sustainable world [3].

In particular, pineapple (*Ananas comosus* L.) industrialization is known to generate a significant amount of solid residues (ca. 75%; *w*/*w*) [3]. This raw material is rich in value-added products with several biological activities associated with several molecules, such as, polyphenols, carotenoids [4], and proteolytic enzymes—bromelain (EC 3.4.22.33) [5], but also different type of sugars (smaller and bigger in molecular size) that may be used in fermentation for the production of different ingredients, such as production of ethanol and nanocellulose, beyond other ingredients and applications [3,6,7,8,9,10].

In this context, recent studies have suggested that juices produced from juices can be employed as a substitute medium for the incorporation of probiotics, due to larger amounts of simple sugars, which will stimulate probiotics growth [11]. The major carbohydrates present in pineapple fruit are the low molecular weight sugars, such as glucose, sucrose, and fructose [12], on the other hand, the major organic acids are citric and malic, whereas the content of these acids dramatically changes with different environmental conditions. Salunkhe and Kadam (1995) [13] has described that pineapple juice contains neutral polysaccharides composed predominantly by soluble oligosaccharides, such as galactomannans, while the recognized prebiotic structures are other oligosaccharides, such as fructo-oligosaccharides (FOS), galacto-oligosaccharides (GOS), lactulose, and inulin, there are still a variety of alternative carbohydrates that might display prebiotic potential, amongst these galactomannans.

Galactomannans are polysaccharides with a heterogenous structure spread in nature, and generally possess the main chains made of (1–4)-linked D-mannopyranose (Mannose) units to which are connected (1–6)-linked D-galactopyranose (Galactose) units [14], however, the monomer ratio will differ depending on the original source and to be considered galactomannans, the mannans should contain more than 5% of galactose. The differences in Man/Gal ratios leads to differences in chemical properties, such as, holding water capacity, thickening, gelling, binding, suspending, emulsifying, as well as, film formation, making these molecules suitable for different applications [14].

In this study, the preparation of pineapple juices from by-products (peels and stems) using non-invasive methods was investigated. The novelty lies that before assessing the prebiotic potential of pineapple by-products liquid fraction (PBLF), the fraction containing enzymes was removed from the end juices. The enzymatic fraction removal decreased in the high extension of the proteolytic activity of pineapple juices and increased consequently, the prebiotic potential.

Thus, this work provides an experimental assessment on the prebiotic properties of PBLF and their galactomannans by an in vitro fermentation assay, where PBLF fractions were evaluated prior, through and after simulation of gastrointestinal tract (GIT) for prebiotic activity and carbohydrate composition.

## 2. Materials and Methods

### 2.1. Standards and Consumables

The carbohydrates standards for identification and quantification of oligosaccharides were d-xylose, xylobiose, xylotriose, xylotrehalose, and xylopentaose were acquired at Megazymes (Bray, Ireland), while polysaccharides standards for molecular weight were bought from Shodex^TM^ (Munich, Germany) and d-galactose and d-cellobiose from Sigma-Aldrich (St. Louis, MA, USA).

All the consumables and enzymes used for the simulation of the gastrointestinal tract used in this work were purchased from Sigma-Aldrich (St. Louis, MA, USA): α-amylase, bile salts, pork pancreatin, pork pepsin, as well as hydrochloric acid (HCl), sodium hydrogen carbonate (NaHCO_3_), and sulphuric acid. From Merck (Kenilworth, NJ, USA), potassium persulfate (K_2_S_2_O_8_) was obtained.

For the assessment of antioxidant activities and total polyphenolic compounds, the following reagents were used: Folin-Ciocalteu reagent, fluorescein, ABTS, AAPH, and Trolox and were purchased from Sigma-Aldrich (St. Louis, MA, USA).

The prebiotic assessment was performed through microorganisms’ fermentation using adequate media Muller–Hinton (MH) and de Man–Rogosa–Sharp (MRS) were acquired from Biokar (Allonne, France), while cysteine used as a supplement for MRS was obtained from Merck (Darmstadt, Germany). The positive control used FOS, which was purchased from Orafti (Oreye, Belgium).

### 2.2. Raw Materials

*Ananas comosus* Merr. (pineapples) were exported to Portugal and acquired from Costa Rica at the fresh stage and at ¾ stage of maturation. The pineapple was processed to produced ready-to-eat fruit at NuviFruits—a company belonging to Luis Vicente group from the Portuguese market.

After fruit arrival to the industrial facilities, rotten pineapples were removed and good quality pineapples were disinfected through a washing process, followed by automatically detaching the crown and stem (pineapple core) and automatically cutting off the peel from the remaining fruit. The fruit followed the processing and the parts wasted were immediately frozen at −20 °C for a maximum period of 90 days until further processing.

### 2.3. Samples Preparation

The crude juices were prepared as described previously [15]. The stem and peels (pineapple by-products) were reduced to a crude juice, using a juice machine (model: HR1922/21 1200 W, Philips) that splits the press cake (solid parts) from the crude juice (liquid parts). The crude juice was centrifuged 7370× *g* at 4 °C for 10 min, and the remaining supernatant was centrifuged to remove the remaining pulp.

The enzyme from pineapple fruit is a well-known enzyme in the market, associated with its proteolytic activity and when present in crude juices, influences the prebiotic potential associated, and for that reason an enzymatic extraction was performed to remove the enzymatic fraction from the final product. For that, a green chemistry separation methodology was applied to the remain supernatant as described by Campos et al. [16] to remove bromelain.

The biological activities—prebiotic and antioxidant were assessed for the resulting supernatant (PBLF, without the enzymatic fraction).

#### Samples Preparation for Prebiotic Evaluation

The resulting supernatant from enzyme extraction was frozen at −20 °C, with a cryoprotectant Maltodextrin (MAL 2%; *w*/*v*) and applied to a freeze-drying process through a vacuum pressure of 0.1 torr in the freeze drier (Model FT33, Armfield, UK), and the freezing temperature in the chamber was −46 °C, while the samples chamber temperature was 15 °C.

### 2.4. Prebiotic Activity

#### 2.4.1. Microorganisms’ Growth Conditions

Six commercial probiotic strains were used to evaluate the prebiotic activity of PBLF: *Lactobacillus (L.) rhamnosus* R11 was provided by Lallemand (Montréal, QC, Canada); *Bifidobacterium (B.) animalis* B_0_ supplied by CSK (Ede, The Netherlands); *Bifidobacterium (B.) longum* BG3 was provided by Cell Biotech (Hellerup, Denmark); *Lactobacillus (L.) casei* L01, *Lactobacillus (L.) acidophilus* LA-5^®^, and *Bifidobacterium (B.) animalis* ssp. *lactis* Bb12^®^ supplied by Christian Hansen (HØrsholm, Denmark); and *Escherichia (E.) coli* ATCC 25922 (provided by CBQF microorganisms library). The strains were stored at −80 °C in medium broth containing 30% (*v*/*v*) glycerol.

#### 2.4.2. Inoculum Preparation

Before prebiotic assay, 50 µL of frozen stocks of each strain were inoculated in 5 mL in a static culture with MRS broth (in the case of *Bifidobacterium*, the medium was supplemented with cysteine hydrochloride) under aerobic conditions for the *Lactobacillus* and under anaerobic conditions for *Bifidobacterium*; while for *E. coli*, a static culture, used MH broth under aerobic conditions. All the cultures used were incubated at 37 °C for 18 h.

#### 2.4.3. In Vitro Fermentation Assay

The prebiotic activity was performed as previously mentioned by Gullón et al. [17] and Sousa et al. [18]. The basal medium was prepared with each condition separated. Each bottle was prepared with a positive control using d-glucose at 2% (*w*/*v*) or with FOS at 2% (*w*/*v*) or with PBLF at 2% (*w*/*v*). All were inoculated with a 2% (*v*/*v*) inoculum of each probiotic strain. Inoculum were obtained as previously described. The assay was performed in duplicate.

Aliquots of 250 µL of all conditions to monitor the microbial growth curves were transferred to a 96-well microplate (Thermo Fisher Scientific, Roskilde, Denmark) and the wells were covered with 50-µL autoclave-sterilized liquid paraffin (Merck, Darmstadt, Germany) to avoid the presence of oxygen. The samples were incubated, and cellular growth was monitored by measuring the OD of the cultures at 660 nm at intervals of 60 min.

On the other side, the different glass bottles were all placed in the orbital incubator at the same temperature with stirring at 100 rpm. For the confirmation of fermentable activity, samples were drawn from the media at 0, 2, 6, 8, 12, 24, and 48 h. Growth curves were monitored by enumeration of viable cells and bacteria metabolism was assessed by pH measurement and determination of simple sugars and organic acids by high performance liquid chromatography (HPLC) analysis.

Viable cells were quantified by the drop count method described by Miles et al. [19] wherein 100 µL of sample where collected and diluted using peptone water (concentration at 1 g/L) through serial decimal dilutions and 20 µL of each dilution were plated, in duplicate, on MRS agar (prepared as explained at Section 2.4.2).

All the assays were incubated at 37 °C for 48 h and the pH was determined using a Crison 52-02 (Crison, Barcelona, Spain).

### 2.5. HPLC Determinations

The oligosaccharides molecular weight, simple sugars, and organic acids were analyzed by HPLC using a Beckman & Coulter 168 series and was performed exactly as described by Campos et al. [20]. For the oligosaccharides separation, two columns packed with hydroxylated polymethacrylate-based gel were used, while for the simple sugars and organic acid separation, an Aminex HPX-87H column [20] was used. All the analysis were performed in triplicate for each experiment.

### 2.6. Gastrointestinal Tract Simulation (GIT)

Studies using GIT simulation as previously described by Madureira et al. [21] was used and employed upon PBLF (Pineapple by-products liquid fraction). Briefly, in three independent experiments, 1 g of sample was added in 20 mL of distilled water. The simulated system mimics human conditions, such as temperature, pH, and the specific enzymes for each GIT stage.

After passage through each section—mouth, stomach, and small intestine (duodenal zone)—the total carbohydrates and polyphenols were characterized. The profile for simple sugars, as well as soluble dietary fiber were analyzed and quantified by HPLC. The total amount of phenolic compounds was quantified by the Folin–Ciocalteu method and evaluated through HPLC analysis. The antioxidant capacity (AA) was also determined using ABTS and ORAC assay, as described below.

The exact conditions of mouth, stomach, and small intestine simulation and following dialysis performed are described at Campos et al. [20], a research work previously performed by this same research group.

### 2.7. Antioxidant Capacity Determination (ORAC and ABTS Methods)

The antioxidant capacity of PBLF and progressive fractions (after each GIT step) were measured by ORAC assay according to Dávalos et al. [22] and adapted by Campos et al. [20]. The software used was Fluostar Control version1:32 R2. Concentrations were expressed in µmol Trolox Equivalents (TE)/mL. The same fractions were also evaluated through ABTS radical cation decolorization assay as described by Re et al. [23] and adjusted by Campos et al. [20]. The values were calculated and converted to g/L of ascorbic acid equivalent.

All assays were performed in triplicate, considering three different replicates of the analyzed sample.

### 2.8. Quantification of Total Polyphenols

#### 2.8.1. Folin–Ciocalteu Method

The total content of polyphenols at PBLF was measured using a modified Folin–Ciocalteu method described by Gao et al. [24] and modified by Campos et al. [20]. A standard curve was performed using different concentrations of gallic acid. All measurements were performed in triplicate, for each experiment.

#### 2.8.2. Individual Phenolic Compound Identification by HPLC

The phenolic profile of the PBLF juice was assessed using equipment from Waters e2695 with a separation interfaced module system coupled to a Photodiode array UV/Vis detector (PDA 190–600 nm), according to the method described previously by Campos et al. [25] and later adapted to the pineapple phenolic compounds, as describe by Campos et al. [15].

A 20-µL injection was used and detection was performed ranging from 200 to 600 nm wavelengths, measured in 2-nm intervals. Peaks were searched by type, namely: Catechins/procyanidins were searched at 280 nm, phenolic acids at 320 nm, flavonols at 330 nm, and anthocyanins at 520 nm. For confirmation, the retention times and spectra of pure standards were used. Three independent analyses were performed for each experiment.

### 2.9. Statistical Analysis

The homoscedasticity assumption was met through analysis of variance (ANOVA), using a 95% confidence interval, and was applied to each dependent parameter. The Tukey test, with a 95% confidence interval, was used for pair-wise comparisons. Correlation between the parameters was determined by Spearman’s rho, with a 99% confidence interval. All the statical analysis was performed using v. 500 software, GraphPad Prism (GraphPad Software, San Diego, CA, USA).

The prebiotic effect evaluation was performed by comparing the obtained data of the tested samples against the positive and negative controls for the studies strains. The slope determination was used to calculate the OD_660_ trend line through each growth curve log phase [18].

## 3. Results

### 3.1. Structural Features and Chemical Composition of Pineapple By-Products

The molecular weight (Mw) of oligosaccharides, linkage, and monosaccharide composition have an important role in the oligosaccharide’s biological effects [26]. Separation and identification of simple sugars was performed by the HPLC method, being identified as main sugars d-glucose and fructose, differences were found in the proportion of the identified sugars, in the different samples.

The polysaccharides analysis was performed also by HPLC, calibration curves were for monosaccharide, oligosaccharides, as well as polysaccharides. The PBLF (peels and stems) were evaluated and two major peaks of 2000 Da and 600 Da were identified. The Mw of the identified structure are typically from oligosaccharides. Salunkhe et al. [13] have described the presence of oligosaccharide in pineapple juice and have identified galactomannans in very small dimensions.

### 3.2. Effect of Pineapple By-Products on the Prebiotic Growth

**Figure 1:**
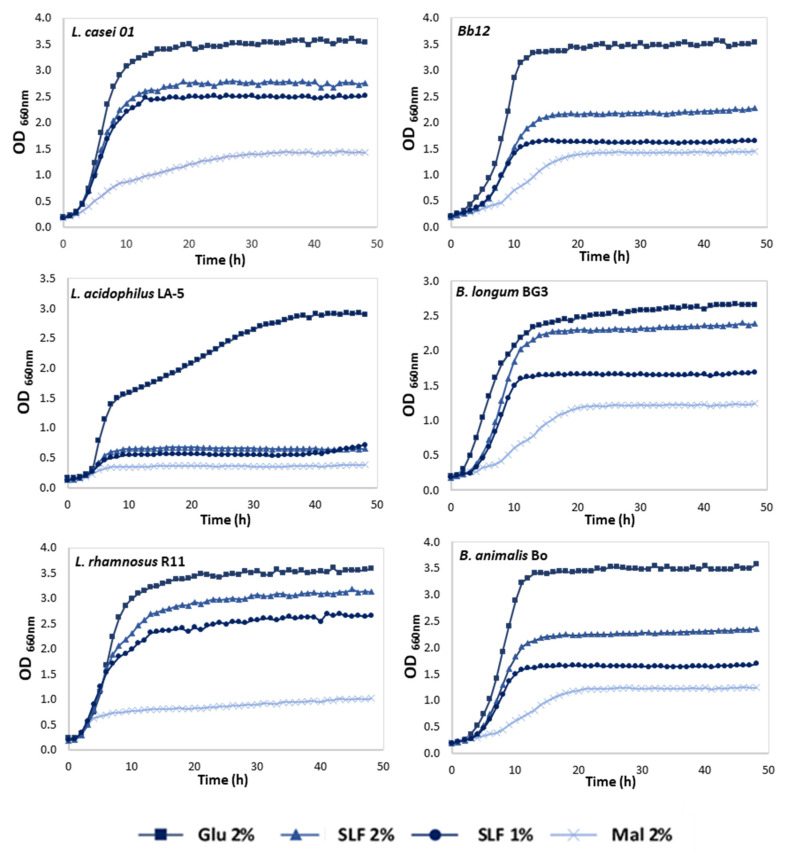
Growth curves of lactobacilli and bifidobacteria strains in media containing glucose (Glu) or maltodextrin (Mal) at 2% (*w*/*v*) as positive controls and pineapple stem liquid fraction (PSLF) at 2 or 1% (*w*/*v*).

To evaluate the suitability of pineapple by-products as a fermentable carbohydrate source for probiotic bacteria, an important pre-condition should be the full field and understanding if PBLF are able to metabolize as well, or nearly as well, as glucose by a specific strain [27]. Thus, in vitro screenings were carried out using six different strains, three *Lactobacillus* (*L. acidophilus*, *L. casei*, and *L. rhamnosus*) and three *Bifidobacterium* (Bb12, B_0_, and BG3), and the growth of all strains were controlled every hour over 48 h. The growth profiles of the representative strains are shown in Figure 1.

The results showed a limited growth occurred for negative control, as expected, confirming that the growth enhancement of the samples with PBLF was a consequence of the presence of carbohydrates from the tested fraction.

Maltodextrin was used at 2% (*w*/*v*) in the PBLF for improvement of the freeze-drying process, therefore growth curves for this compound were also performed to study the effect of this carbohydrate upon the studied probiotic microorganism. Moreover, based on maximum growth rates achieved (Table 1), maltodextrin at 2% (*w*/*v*) did not present growth capacity in all the tested strains.

**Figure 2:**
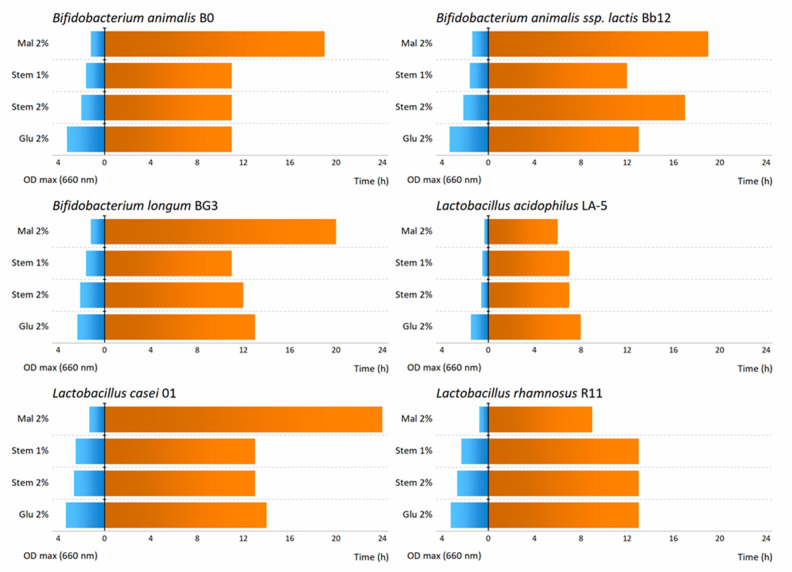
Maximum optical densities (OD660) (at left in blue) versus at corresponding fermentation times (at right in orange) using bifidobacteria and lactobacilli strains in media containing glucose (Glu) or Maltodextrin (Mal) at 2% (*w*/*v*) as positive controls and pineapple stem liquid fraction (Stem) at 2 or 1% (*w*/*v*).

Figure 2 presents the maximum OD_660_, as well as the corresponding fermentation time that maximum growth was achieved, and the maximum growth rates for each sample and microorganisms are listed in Table 1. *Lactobacillus casei* followed by R11 exhibited the highest capacity to grow with the PBLF (it presented the maximum µ_max_ values among all the strains), being the highest concentration, 2% (*w*/*v*), which was the best concentration as expected. In addition, when evaluating the impact of positive control in all strains, it was possible to conclude that the fastest growth was also achieved for *L. casei* (glucose, 0.47 h^−1^), as well as for PBLF (0.34 h^−1^), which could mean that the PBLF was used as a carbon source for microorganism’s growth.

Regarding the LA-5, no growth enhancement was observed for PBLF, since low cell density reached a maximum OD_660_ value of 0.647 and 0.718, for 2% and 1% (*w*/*v*), and showed the lowest growth rates (0.09 h^−1^ and 0.07 h^−1^, respectively) of all tested microorganisms (Figure 1).

Regarding to the *Bifidobacterium* strains, an enhancement at growth effect was observed after 48 h using PBLF and maltodextrin as a prebiotic enhancer. In the screenings performed with three different strains, it was possible to conclude that all microorganisms presented similar behaviors regarding PBLF, when analyzing the higher tested concentration, it was possible to see that a similar cell density reached maximum OD (ranging between 2.268–2.385), in addition to, similar maximum growth rates (ranging between 0.24–0.27 h^−1^). Thus, the only difference between the tested *Bifidobacterium* was the growth of positive control (glucose) for BG3, where it presented a growth rate of 0.25 h^−1^, and when compared with the higher concentration of PBLF (0.27 h^−1^), it can be concluded that the microorganisms use PBLF more efficiently than the control, which means that PBLF at 2% (*w*/*v*) could be used as a carbon source and therefore as a prebiotic enhancer.

As described previously in this work, PBLF contains a high concentration of simple sugars (glucose and fructose), which means these compounds can be used as a carbon source by the microorganisms to maintain and therefore enhance probiotic growth. In addition, as described before, it was possible to identify some oligosaccharides in PBLF that could be recognized as galactomannans or glucomannans, as described elsewhere [13]; these type of oligosaccharides could be used to enhance microbial growth in some probiotic strains, using a similar mechanism as the one used in the FOS, a well-known prebiotic activity enhancer; thus a mechanism could be proposed, with simple sugars allowing the initial growth of the microorganisms, but the PBLF oligosaccharides could be used as a carbon source through time, as a second stage source. Our results indicated that pineapple substrates can promote the growth of all tested strains apart from LA-5. Furthermore, some differences in carbohydrate consumption capabilities and growth kinetics by distinct probiotic strains were observed.

Several studies are being performed in order to valorize other type of food by-products, recently, Kurdi and Hansawasdi [28] reported that an oligosaccharide mixture obtained from cassava pulp, a by-product of cassava starch production, had the ability to promote the growth of some tested *Lactobacillus* and *Bifidobacterium* strains.

### 3.3. Fermentation Metabolites—Production of Acetic and Lactic Acids

Prebiotics fermentation by colonic bacteria mainly raises the production of several acids in the large intestine leading to a decline of pH values. The pH turns to a more acidic environment associated with the increase of produces acids at the medium, which allows the growth of lactic acid bacteria, inhibiting the proliferation of potentially pathogenic microorganisms and putrefactive bacteria [29].

In this research work, the prebiotic potential of PBLF is evaluated in an in vitro model. The prebiotic activity was studied for six strains and two concentrations of PBLF, but studies on fermentations profiles were studied for the best strains, one from lactobacilli and other from bifidobacteria (*Lactobacillus rhamnosus* R11 and *Bifidobacterium animalis* B_0_). Therefore, the fermentation was studied through time until 48 h (the values from 2, 6, 8, and 12 h were not included, since no relevant changes were observed). Through HPLC analysis, two simple sugars (glucose and fructose) were identified, as well as four organic acids (lactic, acetic, citric, and formic acid). The production of these organic acids resulted from the specific and different fermentation metabolism of probiotic strains (Table 2).

The *Bifidobacterium* strains are heterofermentative and usually promotes hexose metabolism, producing not only the lactic acid, but also acetic acid [30]. The organic acids profile of *B. animalis* B_0_ showed an increased concentration of lactic acid for the sample regarding PBLF and for positive control (glucose at 2% (*w*/*v*)), which lead to a pH decrease (Table 2). The concentration of formic acid and acetic acid slightly increased throughout 48 h of fermentation. The citric acid presented a different behavior, as expected, with a slight decrease being observed for the positive control with glucose and with FOS. For PBLF the decrease of citric acid in solution was theoretically higher than the positive controls. These results agree with the ones reported by [18], since the slight increase of acetic acid was probably due to the citrate degradation by citrate metabolism. Variations on sugar profile showed that PBLF promoted an increase on concentration of glucose and fructose in the solution until 24 h.

Conversely, between 24 and 48 h, there were a decrease in glucose, but a maintenance of fructose concentration, which demonstrate the metabolization of d-glucose by *B. animalis* B_0_ (Table 2). The positive control with FOS demonstrated a totally different behavior. A small or null initial concentration of sugars was observed, with slight increases during fermentation, proving the metabolization of FOS during fermentation. The behavior also occurred for PBLF, proving the metabolization of more complex sugars (oligosaccharides and polysaccharides present in soluble dietary fiber) into simple sugars, leading to an increase in solution of simple sugars through time, a behavior explained before by Southgate et al. [31]. The sugar and organic acids profiles changes was accompanied by the gradual decrease of pH in all tested samples (Table 2).

Conversely, of *Bifidobacterium* strains, *Lactobacillus* strains are homofermentative organisms and their main organic acid produced is lactic acid, but other acids such as, acetic acid can also be produced from citrate metabolism [30]. Therefore, a pH decrease was expected throughout fermentation. The positive control with glucose demonstrated to have the most extensive pH decrease, followed by PBLF and then FOS. The sugars profile was similar to the ones visualized for *B. animalis* B_0_, however differences were found for FOS because small concentrations of this sugar was quantified. Yet, for PBLF, it was possible to see a decrease of glucose concentration, as well as the disappearance of fructose from the medium. Statistical analysis was performed between variation of sugars and organic acids with a variation of pH. The correlation between glucose and pH through time for FOS was negative (*r =* −0.511), as expected, but such differences were not statistically significant (*p <* 0.05). The lactic acid production was substantial for positive control, followed by FOS at 2%, however the increase for PBLF was not statistically significant (*p <* 0.05). Strangely, the acetic acid concentrations remain stable throughout fermentation for PBLF and the positive control with glucose, the exception was the positive control using FOS. The results for PBLF and glucose were not in accordance with the ones obtained for citric acid production, since positive correlation values between the production of acetic acid and the consumption of citric acid were presented. The decrease of citric acid was not correlated with increasing of acetic acid in the medium. On the other hand, the fermentation with FOS presented a negative correlation (*r =* −0.238) between the values of acetic and citric acid [30]. Formic acid production was detected, and slight differences were found throughout the time for all the tested samples (Table 2).

The present results revealed that PBLF has potential to be applied as a prebiotic enhancer to most of the tested probiotic strains when used as a single carbon source. Moreover, the fermentation resulted in the production of healthy metabolites and seems a good candidate to be incorporated in functional food products, acting as a fermentable carbohydrate source for support of probiotic strains growth. The previous conclusions agreed with a number of studies in which the ability of lactobacilli and bifidobacterial to use different fruit and vegetable sources as substrates for the fermentation of probiotic or colonic microorganism is demonstrated [18,19,20,21,22,23,24,25,26,27].

### 3.4. Phenolic Compounds and Antioxidant Activities

The antioxidant activity, as well as the content of phenolic compounds of PBLF was measured to understand the potential as an antioxidant fraction. Hence, four types of assays were performed to evaluate the maintenance of phenolic compounds and their antioxidant capacity. Table 3 depicts the values of antioxidant activity, measured through ABTS and ORAC assays, as well as the total phenolic content through the Folin–Ciocalteu method and Table 4 shows the phenolic compounds identified and quantified presented in the pineapple stem liquid fraction (PSLF) and pineapple peel liquid fraction (PPLF).

The ABTS colorimetric assay measures the capacity of phenolic compounds to revert the radical cation ABTS to its original state [25]. The values of ABTS obtained for the initial liquid fractions of pineapple by-products were in accordance with the ones exhibited previously by Dávalos et al. [19]. The stem fraction showed lower values of ascorbic acid equivalents for ABTS assay than the peel liquid fraction. Differences found between stem and peel fractions were statistically significant (*p <* 0.05). When the antioxidant capacity was evaluated by ORAC, the results followed the same tendency than the ABTS assay.

However, the values for Trolox equivalents were much higher than ABTS. Campos et al. [22] have described the mechanism action of both assays, whereby several times the ABTS radical is not able to interact with the phenolic compounds of biological samples because they are complexed or glycosylated with other structures, such as soluble carbohydrates from dietary fiber [19]. On the other hand, the ORAC assays reflects the peroxyl scavenging activity of the phenolic compounds of samples, being more suitable to evaluate biological antioxidant activity [22]. The initial values of ORAC assay have shown that PSLF presented a higher concentration of equivalents of TE (Trolox) than PPLF. Statistical analysis showed that was not found (*p >* 0.05). When these methodologies were compared, differences within the results were found and, as expected, the ORAC method showed a higher correlation in the results. Other authors have described the same outcome, since ORAC assay is more efficient and trustable than ABTS assay for measuring biological samples [17].

The high increase of antioxidant activity through ABTS throughout GIT simulation shows the release of chlorogenic acid for PPLF and PSLF and the increased concentration of caffeic and ferulic acids at the intestinal phase. The incremented concentrations are associated with the links breaks coupled to the vegetal cellular membrane present at the soluble dietary fiber, where it has been described that larger size phenolic acids have been associated, such as chlorogenic and coumaric acids, which only occur at the duodenal phase, as previously described by Campos et al. [20,32].

The total content of phenolic compounds was evaluated through the Folin–Ciocalteu method, the gallic acid equivalents for both tested liquid fractions. The total content of phenolic compounds, in comparison to the antioxidant activity assays content, was higher for stem liquid fraction, than for peel liquid fraction, with the results presenting significant differences (*p* < 0.05). The identification of phenolic compounds was performed by HPLC, and the graphics were previously published by Campos et al. [20], comparing the PSLF and PPLF with standard phenolic compounds. In the general analysis, some peaks were not identified, but the ones identified for PBLF corresponded to di-hydroxycaffeic, chlorogenic, caffeic, coumaric, and ferulic acid, which are typical phenolic compounds identified for PBLF. Specific phenolic compounds from pineapple fruit identified caffeoyl, feruloyl aldarates, and sinapoyl hexoside. The hydroxycinnamic acids and especially the ferulic acids occurring in the ester-linked to the primary cell walls of pineapple, usually are linked to different polysaccharides, therefore usually feruloyl and caffeoyl oligosaccharides can be identified in pineapple by-products [33,34]. In the literature, other phenolic compounds can be identified for pineapple crudes juices, but in the majority of the works applied, organic solvents were applied in the extractive process to increase the yield of phenolic compounds extraction [35].

### 3.5. Effects of the Simulated GIT upon Pineapple Liquid Fractions

The simulation of GIT was performed with the combination of pH and gastrointestinal enzymes, mimicking the major conditions present in each stage, stomach and small intestine (duodenum). The HPLC analysis was performed to understand the behavior of specific phenolic compounds and carbohydrates profiles throughout GIT. Moreover, the antioxidant capacity, as well as the total phenolic compounds of PSLF and PPLF were also evaluated after each step of simulated GIT and are shown in Table 3 and Table 4.

#### 3.5.1. Effects of GIT Simulation upon Pineapple By-Products Liquid Fractions

The stem liquid fraction showed that antioxidant capacity increased throughout the system, after stomach and after duodenal (small intestine) simulated digestion. However, the same trend was not found in total phenolic compounds. Smaller values of total gallic acid equivalents were found after the intestine stage when compared with the stomach stage. Statistically, significant differences were found for all values of total phenolic compounds for stem liquid fraction (*p >* 0.05).

On the other hand, the results obtained for peel liquid were slightly different for antioxidant capacity measured by ORAC, since the presented values decreased through the GIT, however the values presented for ABTS assay and Folin–Ciocalteu were in accordance with the values present for stem liquid fraction. The total increase of total phenolic compounds, as well as the antioxidant activity after stomach digestion demonstrates that a large amount of phenolic compounds were linked to oligosaccharides and polysaccharide and through this stage these links were cut, releasing more phenolic compounds, being in free state in the solution to be detected and measured through ABTS assay and the Folin–Ciocalteu method. After intestinal digestion, the total amount of phenolic compounds decreases for both pineapple liquid fractions, a behavior also reported by Changpraykaew and Petchlert [36]. However, this was an expected result, since previous characterization worked developed by Campos et al. [32] reported that the major responsible sources for antioxidant activity in pineapple juices were typically compounds usually found in pineapple juice, sinapoyl-glutathione, glutamyl-*S*-sinapyl-cysteine conjugate, and caffeoylquinic acid, identified through UPLC-MS. Due to the intestinal phase digestion, the link of these molecules to other structures, that at the identifying point, are no longer stable. First, because the bile salts tend to bind with the active substances leading to the reduction of total phenolic compounds values detected, and second because of the shift of pH 2 of stomach to pH 7 in the intestine leads to the irreversible breakdown of some phenolic compounds [36,37].

#### 3.5.2. Phenolic Compounds

HPLC analysis (Table 4) showed that stem liquid fraction before simulated GIT presented three quantified phenolic compounds (chlorogenic, caffeic, and ferulic acids) and six identified compounds (caffeoyl and feruloyl aldarates, di-hydroxycaffeic acid, caffeic acid, chlorogenic acid, and ferulic acid). After the mouth stage, the total quantification of phenolic compounds by HPLC analysis increased, followed by a decrease after the stomach stage and a second decrease after the duodenal stage. In the mouth stage, three more molecules were also identified, coumaric acid, *N*-_L_-γ-glutamyl-*S*-sinapyl-_L_-cysteine, and *N*-[(Benzyloxy)carbonyl] leucyileucin amide; besides the decrease of the total amount in the sample, all the phenolic compounds were identified throughout GIT, as can be seen in Table 4. Campos et al. [25] previously described the same behavior when exposing free herbal extracts to simulated GIT.

A similar behavior was found for the peel liquid fraction, where the same three phenolic compounds were quantified and the same six were identified. The initial profile of identification and quantification of phenolic compounds changed throughout GIT stages, a behavior observed for both liquid fractions. After mouth stage, nine phenolic compounds (the previous ones identified, plus eugenin, sinapoyl hexoside and *N*-[(Benzyloxy)carbonyl] leucyileucin amide) were found, against the six phenolic previously identified. After stomach and duodenal stages, there was a total decrease of all identified phenolic compounds. As explained before, the main constitutes of pineapple juices are simple and complex sugars; the phenolic compounds interact with these structures forming links to be more stable. After the mouth stage, the samples were in contact with a different pH, as well as, in contact with the mouth enzyme and salivary amylase. This enzyme starts carbohydrates digestion in the mouth and cleavage complex carbohydrates to smaller chains, or even simple sugars, leading to the release of phenolic compounds in this stage, allowing to be free for detection and quantification. Throughout the stomach stage, there is a drastic decrease in pH and the addiction of porcine pepsin, as supposedly the interaction with this enzyme does not interfere with carbohydrates, but a decrease in total phenolic compounds were found, due to the high low pH. The low pH leads to changes in the phenolic compounds structure or even leads to total loss of such molecules. In the intestinal phase, there is an increase of pH to neutralize the acidity of the stomach and to activate the duodenal pancreatin (mixture of pancreatic enzymes, amylase, lipase and protease) and the bile salts activation. The intestinal stage is very important for lipids digestion because the bile acts as a surfactant by developing micelles to increase the available surface of lipids to be digested by the pancreatic lipase, moreover the pancreatic protease is also responsible for the breakdown of protein into amino acids and the pancreatic amylase is responsible for the breakdown of starch and glycogen, leading to the release of simple sugars and other structures linked to these molecules, such as polyphenols. The results have showed a decrease in the quantified phenolic compounds, which means that at this point there was no release of other phenolic compounds complexed with carbohydrates or proteins, and the remaining ones were degraded by the action of the duodenal medium. Other authors have reported the same behavior, a significant decrease of free polyphenols when exposed to simulated GIT, leading to a general decrease of antioxidant activity of tested samples [25,38]. 

### 3.6. Simple Sugars and Complex Carbohydrates

**Figure 3:**
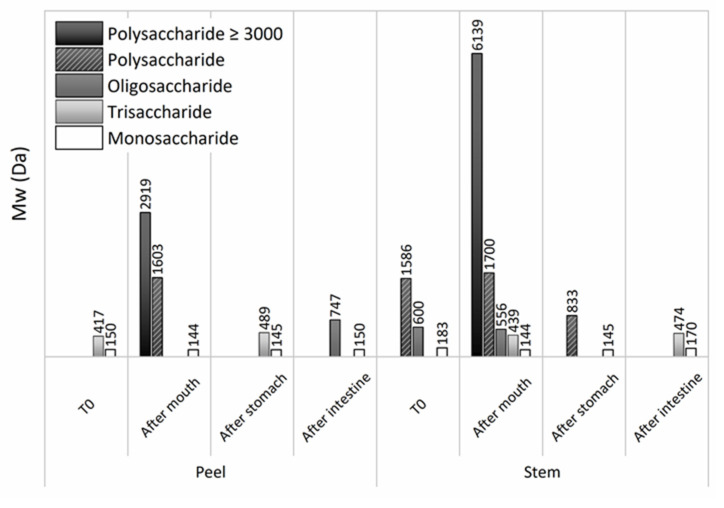
Soluble carbohydrates molecular weight assessment (mean ± standard deviation) along GIT simulation. Samples were taken after mouth, stomach and intestine for both studied pineapple by-product liquid fractions (peels and stems).

The molecular weight of carbohydrate profile was also evaluated by HPLC analysis (Figure 3). The samples before simulated GIT have shown the same profile that is previously reported in this work.

Both pineapple liquid fractions have shown a differentiation after each GIT stage, showing a decrease of polysaccharide towards smaller carbohydrates, such as, trisaccharide, disaccharide, and monosaccharides. After the mouth stage for both by-products, an appearance of polysaccharides occurred that were not present in the initial sample, which is probably because these polysaccharides were complexed with other molecules, having higher molecular weight. However, after the mouth stage, as explained before, the carbohydrate digestion starts by action of mouth amylase, leading to the appearance of polysaccharide of 3000 and 1600 kDa for PPLF, while for PSLF, polysaccharides of 6000 and 1700 kDa were found. Sanchez et al. [39] also described the digestion of arabinoxylan-oligosaccharides through fermentation and results indicated that such carbohydrates can be digested through GIT simulation by action of pH and gastrointestinal enzymes.

**Figure 4:**
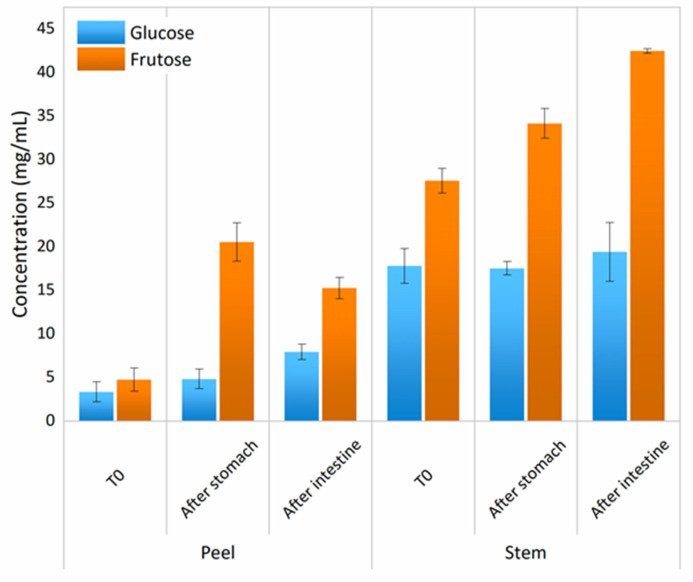
Evaluation content of glucose and fructose (mean ± standard deviation) through GIT simulation. Samples were taken after mouth, stomach, and small intestine (duodenal) stages for both studied pineapple by-product liquid fractions (peels and stems).

In the next stage of GIT, a decrease of carbohydrates Mw was noticed, being only detected in the presence of tri-, di-, and monosaccharides, which means total carbohydrates digestion in the solution, from dietary fiber into simple sugars, as expected. The presented results were also corroborated by the ones depicted in Figure 4—a HPLC analysis for detection of simple sugars throughout GIT simulation.

Results showed that during GIT simulation for PBLF there was a maintenance of d-glucose in solution, but a high increase of fructose. Differences between stages showed to be statistically significant for d-glucose (*p >* 0.05). The PBLF showed an increase of both simple sugars, being once more the highest release for fructose. Differences were not found for glucose release, in contrast to fructose release. When comparing pineapple fractions PPLF and PSLF, differences statistically significant were found (*p >* 0.05). The concentration of simple sugars was much higher for PSLF, being in accordance with the evaluation by HPLC of digested carbohydrates, reported in Figure 3, which showed a higher transition of polysaccharides into monosaccharides.

#### Prebiotic Activity

Prebiotic activity was also evaluated throughout the simulated GIT, after mouth, after stomach, and after duodenal stages. Only five probiotic microorganisms were tested, because *L. acidophilus* LA-5 tested previously did not show prebiotic activity upon PBLF.

**Figure 5:**
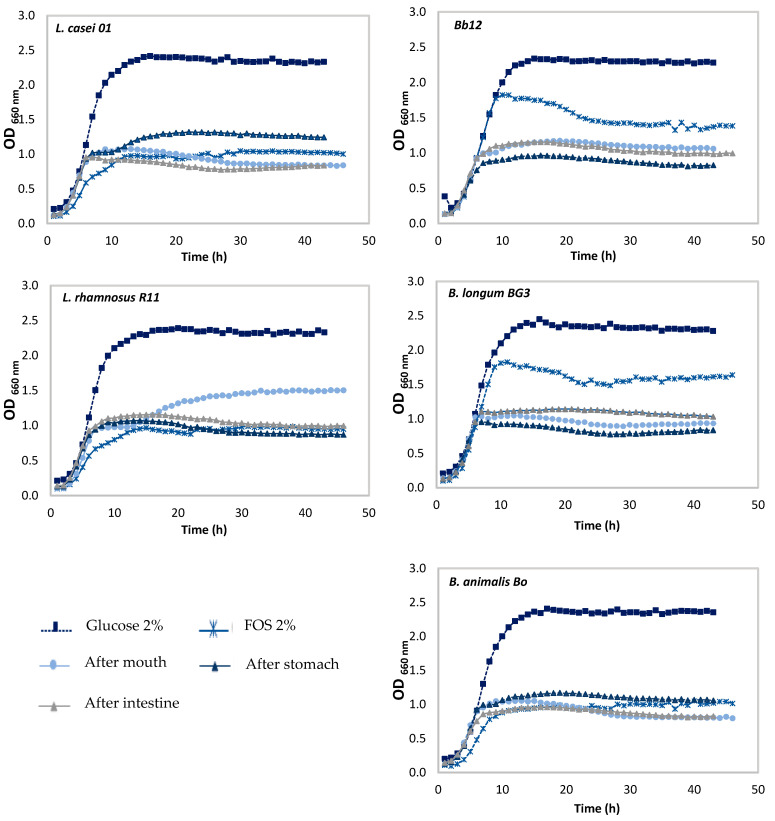
Evaluation of growth curves of lactobacilli and bifidobacteria strains in media containing glucose or frutooligosaccharides (FOS) at 2% (*w*/*v*) as positive controls, and pineapple stem liquid fraction (PSLF) along the simulation of GIT.

**Figure 6:**
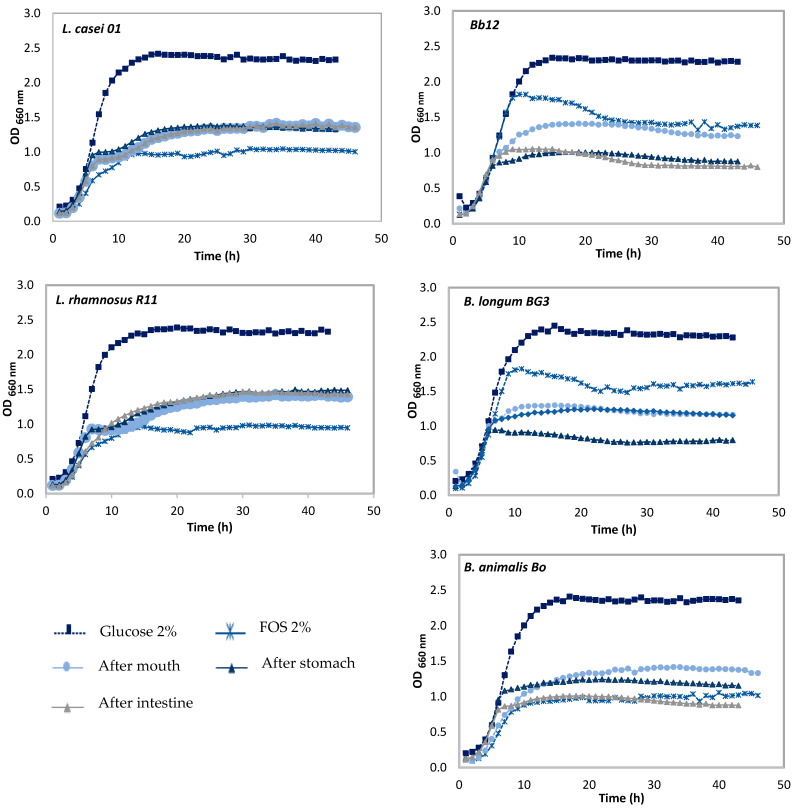
Evaluation of growth curves of lactobacilli and bifidobacteria strains in media containing glucose or frutooligosaccharides (FOS) at 2% (*w*/*v*) as positive controls, and pineapple peel liquid fraction (PPLF) along the simulation of GIT.

The evaluation of prebiotic activity of stem liquid fraction for all probiotic strains are shown in Figure 5 and for peel liquid fraction are shown in Figure 6. Comparisons with Figure 1 (initial prebiotic activity) were made for with each GIT stage simulation.

PSLF differences were found after the mouth stage when compared with the initial result. A high decrease of prebiotic activity upon all tested probiotic microorganisms was found, with the exception of *L. rhamnosus* R1. Although, when comparing the gastrointestinal stage, small variations were found, but the differences were not statistically significant (*p <* 0.05).

Similar behavior was found for peel liquid fraction, since there was a decrease of prebiotic activity after the mouth stage for all probiotic strains, with the exception of *L. rhamnosus* R11 and Bb12. These was not an expected result because the previous analysis of HPLC showed the increased release of simple sugars during GIT simulation. However prebiotic activity using biological samples is not a linear activity, since several factors account to such potentiality, such as the presence of other molecules that could act against prebiotic activity, such as the presence of polyphenols, which could act as antimicrobial agent [39,40].

*L. casei* 01 did not show any differences between the gastrointestinal stages, on contrary of the remain strains. Differences were found for Bb12 and *B. longum* BG3, between the mouth stage and duodenal stage, which means digestion somewhat negatively affected the prebiotic action upon the probiotic strains. For the Lactobacilli strains, differences between the gastrointestinal stages were not found.

Through these results, it is possible to conclude that both tested liquid fractions presented different prebiotic activity behavior towards GIT simulation. PPLF showed to be a better enhancer than the stem liquid fraction, since slightly higher growth was found for the same probiotic strains, but the differences were not statistically significant (*p <* 0.05).

As a conclusion, the mimicking of GIT negatively affected the prebiotic activity of all strains, with differences being statistically significant (*p >* 0.05). These were not an expected result, since through HPLC analysis it was possible to detect and identify the presence of simple sugars (glucose and fructose) throughout all the GIT stages, being the most representative of the mouth stage. Moreover, the analysis of polysaccharides Mw showed that pineapple polysaccharides and oligosaccharides (galactomannans) were digested throughout simulation generating tri-, di-, and monosaccharides, which can be used by probiotic strains as an energy source. On the other hand, and as described before pineapple oligosaccharides—galactomannans once digested will release galactose-oligosaccharides and mannan-oligosaccharides, which could not be used as an energy source [38]. Of course, other medium characteristics could negatively affect the growth rate of strains, such as a high pH, and the presence of certain organic acids or polyphenols [39,40].

## 4. Conclusions

Based on the above considerations, it is possible to conclude that pineapple by-products liquid fraction has the potential to be applied as a prebiotic enhancer, since it metabolizes the carbohydrates present in the solution. Pineapple by-products liquid fraction promoted the growth enhancement of probiotic bacteria and increased the production of organic acids. However, during GIT simulation, which began at the mouth stage, led to the release of polyphenols with a high antioxidant capacity, and digestion of typical pineapple galactomannans into simple sugars.

The prebiotic activity did not improve through digestion of GIT, in reality the prebiotic potential decreased, which could mean one of two situations. First, the amount of simple sugars was not enough to be used as a carbohydrate energy source or second, the amount of phenolic compounds released led to the opposite expected effect. Several authors have described the potential of polyphenols as natural antimicrobials and in a certain amount could act as a growth static, and when also applied in a higher amount, could lead to a microorganism’s total inhibition.

The current results helped to understand the direct effect of simulated GIT into pineapple by-products liquid fraction and how the prebiotic and antioxidant activities were affected by GIT conditions. Of course, this work intends to give an overview of potential applications of pineapple by-products, opening the door for the future application and re-incorporation in the food supply chain.

## Figures and Tables

**Table 1:** Maximum growth rates (µ_max_, h^−1^) of six different probiotic strains grown in the presence of freeze-dried pineapple by-products juice extract (1 and 2%) compared with control.

Probiotic Strains	Maximum Growth Rate (µmax/h)			
	Positive Control (*w*/*v*)		Pineapple Extract (*w*/*v*)	
	GLU 2%	MAL 2%	2%	1%
*Bifidobacterium animalis* B0	0.4347	0.0682	0.2587	0.2022
*Bifidobacterium animalis* ssp. *lactis* Bb12	0.4725	0.0960	0.2425 ^a^	0.2011 ^a^
*Bifidobacterium longum* BG3	0.2511 ^a^	0.0679	0.2646 ^a^	0.1955
*Lactobacillus acidophilus* LA-5	0.2840	0.0340	0.0877 ^a^	0.0664 ^a^
*Lactobacillus casei* 01	0.4748	0.0952	0.3366 ^a^	0.3120 ^a^
*Lactobacillus rhamnosus* R11	0.4069	0.0383	0.3198	0.2652

Abbreviations: GLU—glucose; MAL—Maltodextrin. ^a^ The means with superscript at the same row are not statistically significant (*p* > 0.05). Analysis of variance was used to estimate the effects of each carbohydrate percentage in the microbial growth of different probiotic strains. Tukey test was used as post-test. The expressed values are the equation slope (m), means maximum growth rate.

**Table 2:** Differences of the pH value, fructose, glucose, lactic, acetic, citric, and formic acids concentrations, for the two probiotic strains tested in the different MRS culture media.

Probiotic Strains	Parameter	Incubation Time (h)	Positive Controls (*w*/*v*)		Stem 2% (*w*/*v*)
			GLU 2%	FOS 2%	
*Bifidobacterium animalis* B0	pH value	0	5.97 ± 0.00	6.80 ± 0.03	6.78 ± 0.01
		24	6.00 ± 0.00	6.20 ± 0.01	6.08 ± 0.02
		48	4.23 ± 0.01	5.92 ± 0.01	5.83 ± 0.00
	Glucose	0	2.67 ± 0.04	0.55 ± 0.01	0.53 ± 0.01
		24	3.32 ± 0.01	0.85 ± 0.01	3.36 ± 0.01
		48	1.54 ± 0.01	0.80 ± 0.00	2.12 ± 0.04
	Fructose	0	0.77 ± 0.01	0.00 ± 0.00	0.13 ± 0.05
		24	2.60 ± 0.04	0.69 ± 0.00	2.67 ± 0.04
		48	2.29 ± 0.01	1.00 ± 0.02	2.73 ± 0.09
	Lactic acid	0	0.45 ± 0.03	0.24 ± 0.02	0.07 ± 0.00
		24	0.46 ± 0.01	0.26 ± 0.01	0.47 ± 0.01
		48	5.82 ± 0.08	0.31 ± 0.02	5.41 ± 0.02
	Acetic acid	0	1.64 ± 0.07	1.74 ± 0.12	1.40 ± 0.25
		24	1.64 ± 0.00	1.85 ± 0.00	1.66 ± 0.01
		48	2.02 ± 0.01	1.80 ± 0.03	1.73 ± 0.01
	Citric acid	0	0.77 ± 0.06	1.04 ± 0.04	0.87 ± 0.00
		24	0.73 ± 0.01	0.98 ± 0.01	0.75 ± 0.00
		48	0.74 ± 0.00	0.95 ± 0.01	0.68 ± 0.00
	Formic acid	0	0.21 ± 0.01	0.03 ± 0.00	0.10 ± 0.00
		24	0.24 ± 0.02	0.16 ± 0.03	0.24 ± 0.01
		48	0.20 ± 0.03	0.27 ± 0.06	0.12 ± 0.03
*Lactobacillus rhamnosus* R11	pH value	0	5.98 ± 0.01	6.82 ± 0.02	6.59 ± 0.00
		24	6.04 ± 0.00	6.11 ± 0.01	5.53 ± 0.01
		48	4.41 ± 0.01	5.68 ± 0.01	5.44 ± 0.01
	Glucose	0	1.15 ± 0.07	0.07 ± 0.00	1.15 ± 0.08
		24	3.36 ± 0.01	0.05 ± 0.00	1.01 ± 0.00
		48	2.12 ± 0.04	0.03 ± 0.00	0.48 ± 0.02
	Fructose	0	1.51 ± 0.16	0.01 ± 0.00	1.51 ± 0.16
		24	2.67 ± 0.04	0.25 ± 0.00	0.00 ± 0.00
		48	2.73 ±0.09	0.24 ± 0.00	0.00 ± 0.00
	Lactic acid	0	1.02 ± 0.01	0.10 ± 0.01	1.02 ± 0.01
		24	0.47 ± 0.01	0.75 ± 0.01	1.24 ± 0.01
		48	5.41 ± 0.02	0.72 ± 0.04	1.19 ± 0.01
	Acetic acid	0	1.96 ± 0.02	1.40 ± 0.02	1.96 ± 0.02
		24	1.66 ± 0.01	2.13 ± 0.01	2.04 ± 0.00
		48	1.73 ± 0.01	2.14 ± 0.01	1.91 ± 0.03
	Citric acid	0	0.79 ± 0.02	0.90 ± 0.01	2.95 ± 0.96
		24	0.75 ± 0.02	0.17 ± 0.01	0.15 ± 0.02
		48	0.68 ± 0.00	0.16 ± 0.00	0.12 ± 0.00
	Formic acid	0	0.47 ± 0.09	0.15 ± 0.01	0.47 ± 0.09
		24	0.24 ± 0.01	0.19 ± 0.03	0.24 ± 0.01
		48	0.15 ± 0.03	0.28 ± 0.04	0.24 ± 0.02

Abbreviations: FOS—frutooligossacharides. Sugars and organic acids are presented in mg/mL.

**Table 3:** Evaluation of antioxidant capacity (ABTS and ORAC methods) and total phenolic compounds (Folin–Ciocalteu method) of pineapple by-products liquid fraction (stems and peels) in the simulated GIT. All results are expressed in mg/100 g on dry basis.

Pineapple By-Product	GIT Stage	ABTS Assay (mg AAE/100 g)	ORAC Assay (mg TE/100 g)	Folin–Ciocalteu (mg GAE/100 g)
Peel liquid fraction	T_0_	430.9 ± 9.2	21.7 ± 0.6	476.1 ± 10.3
	After stomach	352.8 ± 12.9 ^a^	14.7 ± 0.5 ^a^	783.9 ± 21.3 ^a^
	After intestine	883.5 ± 24.0 ^a^	16.2 ± 0.3 ^a^	680.3 ± 27.7 ^a^
Stem liquid fraction	T_0_	369.6 ± 8.9	19.5 ± 1.4	529.7 ± 16.6
	After stomach	452.1 ± 27.7 ^a^	25.9 ± 1.8 ^a^	708.8 ± 7.3 ^a^
	After intestine	826.3 ± 15.0 ^a^	29.3 ± 0.7 ^a^	590.7 ± 39.7 ^a^

Abbreviations: Ascorbic acid equivalent, AAE; Trolox equivalent, TE; Gallic acid equivalent. GAE; Gastrointestinal tract, GIT. T_0_—before gastrointestinal tract. ^a^ The differences between the means of T0 with the means in the same column labelled with same superscript are statistically significant (*p* < 0.05). Analysis of variance was used to estimate the effects of GIT upon pineapple liquid fractions.

**Table 4:** Polyphenol’s quantification in pineapple liquid fraction from by-products (stems and peels) during GIT simulation. All results expressed in mg/100 g on dry basis.

Pineapple By-Product	GIT Stage	Chlorogenic Acid (mg/100 g DB)	Caffeic Acid (mg/100 g DB)	Coumaric Acid (mg/100 g DB)	Ferulic Acid (mg/100 g DB)
Peel liquid fraction	T_0_	16.96 ± 1.42	34.54 ± 4.46	0.00 ± 0.00	26.68 ± 3.80
	After stomach	21.28 ± 0.68	12.60 ± 1.4	0.00 ± 0.00	2.68 ± 0.12
	After intestine	133.58 ± 12.44	11.46 ± 0.76	0.00 ± 0.00	0.50 ± 0.44
Stem liquid fraction	T_0_	106.96 ± 1.9	14.62 ± 0.58	0.00 ± 0.00	0.00 ± 0.00
	After stomach	26.16 ± 0.72	47.24 ± 1.84	0.00 ± 0.00	13.58 ± 0.34
	After intestine	141.58 ± 0.84	82.16 ± 1.64	43.74 ± 0.72	19.14 ± 0.22

Abbreviations: GIT—Gastrointestinal tract; T_0_—before gastrointestinal tract; DB—dry basis. Data was obtained through HPLC analysis.

## Data Availability

Data is contained within the article.

## References

[1] Gil L.S., Maupoey P.F. (2018). An integrated approach for pineapple waste valorisation. Bioethanol production and bromelain extraction from pineapple residues. J. Clean. Prod..

[2] Schieber A., Stintzing F., Carle R. (2001). By-products of plant food processing as a source of functional compounds—Recent developments. Trends Food Sci. Technol..

[3] Roda A., De Faveri D.M., Giacosa S., Dordoni R., Lambri M. (2016). Effect of pre-treatments on the saccharification of pineapple waste as a potential source for vinegar production. J. Clean. Prod..

[4] Ketnawa S., Chaiwut P., Rawdkuen S. (2012). Pineapple wastes: A potential source for bromelain extraction. Food Bioprod. Process..

[5] Dighe N.S., Pattan S.R., Merekar A.N., Laware R.B., Bhawar S.B., Nirmal S.N., Musmade D. (2010). Bromelain A Wonder Supplement: A Review. Pharmacologyonline.

[6] Connolly M.L., Lovegrove J.A., Tuohy K.M. (2010). Konjac glucomannan hydrolysate beneficially modulates bacterial composition and activity within the faecal microbiota. J. Funct. Foods.

[7] Mohammad G., Andreas D., Klaus D. (2010). Isolation of polysaccharides from pineapple fruit pulp and their enzymatic liquifaction. Int. Food Res. J..

[8] Omojasola P.F., Jilani O.P., Ibiyemi S. (2008). Cellulase production by some fungi cultured on pineapple waste. Nat. Sci..

[9] Selani M.M., Brazaca S.G.C., dos Santos Dias C.T., Ratnayake W.S., Flores R.A., Bianchini A. (2014). Characterisation and potential application of pineapple pomace in an extruded product for fibre enhancement. Food Chem..

[10] Simas-Tosin F.F., de Souza L.M., Wagner R., Pereira G.C., Barraza R.R., Wendel C.F., Sassaki G.L., Iacomini M., Gorin P.A. (2013). Structural characterization of a glucuronoarabinoxylan from pineapple (*Ananas comosus* (L.) *Merrill*) gum exudate. Carbohydr. Polym..

[11] Ding W., Shah N.P. (2008). Survival of free and microencapsulated probiotic bacteria in orange and apple juices. Int. Food Res. J..

[12] Pruthi J., Girdhari L. (1955). Varietal trials in canning of pineapples. Cent. Food Technol. Res. Inst..

[13] Salunkhe D.K., Kadam S. (1995). Handbook of Fruit Science and Technology: Production, Composition, Storage, and Processing.

[14] Srivastava M., Kapoor V. (2005). Seed galactomannans: An overview. Chem. Biodiver..

[15] Campos D.A., Coscueta E.R., Valetti N.W., Pastrana-Castro L.M., Teixeira J.A., Picó G.A., Pintado M.M. (2019). Optimization of bromelain isolation from pineapple byproducts by polysaccharide complex formation. Food Hydrocoll..

[16] Campos D.A., Woitovich Valetti N., Oliveira A., Pastrana-Castro L.M., Teixeira J.A., Pintado M.M., Picó G. (2016). Platform design for extraction and isolation of Bromelain: Complex formation and precipitation with carrageenan. Process. Biochem..

[17] Gullón P., Gullón B., Cardelle-Cobas A., Alonso J.L., Pintado M., Gomes A.M. (2014). Effects of hemicellulose-derived saccharides on behavior of Lactobacilli under simulated gastrointestinal conditions. Int. Food. Res. J..

[18] Sousa S., Pinto J., Pereira C., Malcata F.X., Pacheco M.B., Gomes A.M., Pintado M. (2015). In vitro evaluation of yacon (*Smallanthus sonchifolius*) tuber flour prebiotic potential. Food Bioprod. Process..

[19] Miles A.A., Misra S., Irwin J. (1938). The estimation of the bactericidal power of the blood. Epidemiol. Infect..

[20] Campos D.A., Coscueta E.R., Vilas-Boas A.A., Silva S., Teixeira J.A., Pastrana-Castro L.M., Pintado M.M. (2020). Impact of functional flours from pineapple by-products on human intestinal microbiota. J. Funct. Foods.

[21] Madureira A.R., Campos D.A., Oliveira A., Sarmento B., Pintado M.M., Gomes A.M. (2016). Insights into the protective role of solid lipid nanoparticles on rosmarinic acid bioactivity during exposure to simulated gastrointestinal conditions. Colloids Surf. B.

[22] Dávalos A., Gómez-Cordovés C., Bartolomé B. (2004). Extending applicability of the oxygen radical absorbance capacity (ORAC−fluorescein) assay. J. Agric..

[23] Re R., Pellegrini N., Proteggente A., Pannala A., Yang M., Rice-Evans C. (1999). Antioxidant activity applying an improved ABTS radical cation decolorization assay. Free Radic. Biol. Med..

[24] Gao X., Ohlander M., Jeppsson N., Björk L., Trajkovski V. (2000). Changes in antioxidant effects and their relationship to phytonutrients in fruits of sea buckthorn (*Hippophae rhamnoides* L.) during maturation. J. Agric. Food Chem..

[25] Campos D.A., Madureira A.R., Sarmento B., Gomes A.M., Pintado M.M. (2015). Stability of bioactive solid lipid nanoparticles loaded with herbal extracts when exposed to simulated gastrointestinal tract conditions. Int. Food Res. J..

[26] Damen B., Verspreet J., Pollet A., Broekaert W.F., Delcour J.A., Courtin C.M. (2011). Prebiotic effects and intestinal fermentation of cereal arabinoxylans and arabinoxylan oligosaccharides in rats depend strongly on their structural properties and joint presence. Mol. Nutr. Food Res..

[27] Huebner J., Wehling R., Hutkins R. (2007). Functional activity of commercial prebiotics. Int. Dairy J..

[28] Kurdi P., Hansawasdi C. (2015). Assessment of the prebiotic potential of oligosaccharide mixtures from rice bran and cassava pulp. LWT Food Sci. Technol..

[29] Vasiljevic T., Shah N.P. (2008). Probiotics—From Metchnikoff to bioactives. Int. Dairy J..

[30] Østlie H.M., Helland M.H., Narvhus J.A. (2003). Growth and metabolism of selected strains of probiotic bacteria in milk. Int. J. Food Microbiol..

[31] Southgate D. (1995). Digestion and metabolism of sugars. Am. J. Clin. Nutr..

[32] Campos D.A., Ribeiro T.B., Teixeira J.A., Pastrana L., Pintado M. (2020). Integral valorization of pineapple (*Ananas comosus* L.) by-products through a green chemistry approach towards added value ingredients. Foods.

[33] Smith B.G., Harris P.J. (2001). Ferulic acid is esterified to glucuronoarabinoxylans in pineapple cell walls. Phytochemistry.

[34] Steingass C.B., Jutzi M., Müller J., Carle R., Schmarr H.G. (2015). Ripening-dependent metabolic changes in the volatiles of pineapple (*Ananas comosus (*L.) *Merr*.) fruit: II. Multivariate statistical profiling of pineapple aroma compounds based on comprehensive two-dimensional gas chromatography-mass spectrometry. Anal. Bioanal. Chem..

[35] Difonzo G., Vollmer K., Caponio F., Pasqualone A., Carle R., Steingass C. (2019). Characterisation and classification of pineapple (*Ananas comosus* [L.] *Merr.*) juice from pulp and peel. Food Control..

[36] Changpraykaew S., Petchlert C. (2015). Digestive effect on antioxidant activity of three commercial fruit juices. Proceedings of the Burapha University International Conference.

[37] Granado-Lorencio F., Olmedilla-Alonso B., Herrero-Barbudo C., Pérez-Sacristán B., Blanco-Navarro I., Blázquez-García S. (2007). Comparative in vitro bioaccessibility of carotenoids from relevant contributors to carotenoid intake. J. Agric. Food Chem..

[38] Sanchez J., Marzorati M., Grootaert C., Baran M., Van Craeyveld V., Courtin C., Broekaert W., Delcour J., Verstraete W., Van de Wiele T. (2009). Arabinoxylan-oligosaccharides (AXOS) affect the protein/carbohydrate fermentation balance and microbial population dynamics of the Simulator of Human Intestinal Microbial Ecosystem. Microb. Biotechnol..

[39] Daglia M. (2012). Polyphenols as antimicrobial agents. Curr. Opin. Biotechnol..

[40] Gyawali R., Ibrahim S.A. (2014). Natural products as antimicrobial agents. Food Control.

